# Sleep and subjective age: protect your sleep if you want to feel young

**DOI:** 10.1098/rspb.2024.0171

**Published:** 2024-03-27

**Authors:** Leonie J. T. Balter, John Axelsson

**Affiliations:** ^1^ Department of Clinical Neuroscience, Karolinska Institutet, Stockholm, 17165, Sweden; ^2^ Department of Psychology, Stress Research Institute, Stockholm University, Stockholm, 11419, Sweden

**Keywords:** sleep, subjective age, experimental sleep restriction, sleepiness

## Abstract

The current studies examined the impact of insufficient sleep and sleepiness on the subjective experience of age. Study 1, a cross-sectional study of 429 participants (282 females (66%), 144 males, 3 other gender; age range 18–70), showed that for each additional day of insufficient sleep in the last 30 days, subjective age increased by 0.23 years. Study 2, an experimental crossover sleep restriction study (*n* = 186; 102 females (55%), 84 males; age range 18–46), showed that two nights of sleep restriction (4 h in bed per night) made people feel 4.44 years older compared to sleep saturation (9 h in bed per night). Additionally, moving from feeling extremely alert (Karolinska Sleepiness Scale (KSS) score of 1) to feeling extremely sleepy (KSS score of 9) was associated with feeling 10 years older in both studies. These findings provide compelling support for insufficient sleep and sleepiness to exert a substantial influence on how old we feel, and that safeguarding sleep is probably a key factor in feeling young.

## 1. Introduction

Do we not all want to feel young and rejuvenated? Recent findings suggest that this aspiration may hold valuable benefits in defying the passage of time and ageing. Feeling young has been related to brain health, as evidenced by research showing that older adults who feel younger have a younger predicted brain age and larger grey matter volumes in several brain regions [1]. In the light of this connection, we set out to assess whether sleep, a fundamental process crucial for brain function and overall well-being [2], holds any secrets to feeling young.

Subjective age, often referred to as how old we feel, is a concept that goes beyond mere perception. It turns out that feeling younger than our actual age is associated with living longer [3], better mental and physical health [4,5], and more positive psychological traits (e.g. optimism, hope, resilience) [4], prompting suggestions to include subjective age as a biophysical marker of ageing as part of health examinations [6].

Many people feel younger than their calendar age. While this phenomenon is less prevalent among younger individuals, as we reach our thirties and beyond, the discrepancy between how old we are and how old we feel becomes more pronounced [7]. Emerging research suggests that our sleep habits may play a role in shaping how old we feel [6,8]. Sleep is not only crucial for physical well-being but also for mental health. During sleep, the brain undergoes essential processes, including clearance of metabolic waste products [9], filling up of proteins in synapses [10] and memory consolidation [11]. Although it is undisputed that sleep is central for health, its relevance for how young someone feels has received limited attention. There is some support for people that feel subjectively older to have worse sleep quality or sleep difficulties, both cross-sectionally [8] and longitudinally [6], although this may be largely limited to elderly women [8]. Sleep is a highly dynamic process, with gradual alterations across the lifespan, but it also varies on a day-to-day basis. Recognizing the pivotal role of sleep in well-being, we conducted two studies—one cross-sectional and one experimental—to examine how sleep during the last month and more acute sleep loss influence subjective age. Exploratory analyses were performed to assess the influence of chronotype on the relationship between sleep and subjective age.

## 2. Methods

### (a) Study 1: lifespan

#### (i) Participants

This cross-sectional study involved 429 participants (*M*_age_ = 42.0, s.d. = 14.0; 282 (66%) females, 144 males, 1 non-binary, 1 queer, 1 none), who were recruited via the online recruitment platform Prolific.co. Participants had to be fluent in English and resident in the UK, and had to fit one of the following age groups (aiming to have at least 80 participants in each group): 18–30 (*n* = 84), 31–40 (*n* = 85), 41–50 (*n* = 86), 51–60 (*n* = 93), 61–70 (*n* = 81) years of age. Participants provided digital informed consent.

#### (ii) Procedures

All measures were completed via the participant's personal mobile smartphone between 11.00 and 17.00. Data collection took place in June 2022.

#### (iii) Measures

Subjective age was assessed using a single-item question: ‘On some days you may feel older or younger than your calendar age. What age do you feel right now?’. Subjective age was restricted to a lower limit of 12 years and an upper limit of 120 years. The Karolinska Sleepiness Scale (KSS) was used to assess sleepiness. This 9-point scale is a well-validated scale linked to physiological indicators of sleepiness and is strongly affected by sleep [12]. Insufficient sleep was evaluated through a single-item question asking: ‘During the past 30 days, for about how many days have you felt you did not get enough rest/sleep?’. The response option ranged from 0 to 30 days.

The reduced Morningness–Eveningness Questionnaire (rMEQ) [13] was used to estimate chronotype. The total score, ranging from 4 to 25, categorizes individuals into evening chronotypes for scores of 11 or lower, morning chronotypes for scores of 18 or higher, and scores in between as intermediate chronotypes.

#### (iv) Statistical approach

Univariate fixed effect regression models were fitted using the lm function from the stats R package to examine the association between the sleep measures (sleepiness and insufficient sleep) and subjective age. Age deviation was calculated by subtracting calendar age from subjective age, where higher values indicate a subjective age that is older than the individual's calendar age. For the remainder of the text, we refer to age deviation as subjective age. The alpha-level of significance was *p* < 0.05. Exploratory analyses assessed the influence of chronotype on the relationship between sleep and subjective age. Adjustment for age was performed, considering that chronotype has been reported to change with age [14]. For the gender analysis, three individuals who did not identify as either female or male were excluded as three datapoints are deemed insufficient for a valid analysis.

### (b) Study 2: experimental sleep restriction

#### (i) Participants

This ongoing randomized crossover experiment involved 186 participants (*M*_age_ = 25.6, s.d. = 5.3, range 18–46, 102 (55%) females, 84 males), who were invited to complete two sleep conditions: sleep saturation (2 days with 9 h in bed per night, *n* = 173) and sleep restriction (2 days with 4 h in bed per night, *n* = 179). Exclusion criteria were a habitual sleep need outside 7–9 h, a current sleep or psychiatric disorder, having worked night shifts or travelled three or more time zones in the three weeks prior to each session, problems with abstaining from coffee, tea or other caffeine-containing products for 1 day, and lack of fluency in either Swedish or English. Participants provided written informed consent.

#### (ii) Procedures

The test protocol started at least 7 days prior to the laboratory visit. After an individual was deemed eligible through an online screening questionnaire, a familiarization session was scheduled. During this session, participants provided written informed consent, completed questionnaires including the rMEQ, practised tests and received information regarding the sleep protocol. The sleep protocols were carried out in the participants' own beds at home, and sleep timings were based on the individual's habitual sleep timings. The participant and research leader determined the exact sleep timings together to ensure that sleep schedules would not considerably deviate from their habitual sleep timings. Following the familiarization session, there was a 7-day period during which participants kept an electronic sleep diary and wore an actigraph (ActTrust 2, Condor Instruments, Brazil) to facilitate protocol adherence and gather information about physical activity (actigraph data are not analysed here). For the purpose of this study, we have used the time-stamped sleep diary data to assess adherence to the sleep protocol. During the last two nights of this 7-day period, participants slept according to the assigned sleep protocol. The laboratory visits were either completed in the morning (with a start time between approx. 8.30 and 9.30) or in the afternoon (start time between approx. 12.00 and 13.00). To ensure a balanced representation of morning and evening chronotypes across the morning and afternoon sessions, the assignment to either the morning or afternoon session was determined quasi-randomly based on the participant's self-reported chronotype, assessed using a single-item question during the online screening. Participants completed both laboratory visits either in the morning or in the afternoon. Data collection took place between March 2022 and June 2023.

Participants were instructed to avoid naps, strenuous physical activities, abstain from alcohol and not consume caffeinated products for 24 h prior to the laboratory visit. Participants came fasted for the morning sessions or had abstained from food for at least 4 h prior the afternoon sessions. Breakfast or lunch was provided after a blood sample was taken. The subjective age measure was completed before the blood sample.

#### (iii) Measures

Subjective age, sleepiness and chronotype were assessed in the same way as in Study 1.

#### (iv) Statistical approach

To assess whether sleep condition and sleepiness affect subjective age, mixed-effect models were fitted using the lme4 R package [15]. A random intercept for ID was included to account for individual-level variation in this within-subjects analyses. One subjective age datapoint was removed for being more than five standard deviations above the mean (the datapoint was 8 s.d. above the mean). The alpha-level of significance was *p* < 0.05. Exploratory analyses examined the influence of chronotype on subjective age and its potential interaction with sleep condition (adjusted for age). A subset of the participants (*n* = 28) had inaccurately completed the rMEQ item ‘At what time in the evening do you feel tired and as a result in need of sleep?’, probably due to misunderstanding the question or the time choice concerning a.m. and p.m. This specific item was therefore omitted from the rMEQ calculation for these individuals. The rMEQ score for this subset was derived from four items (range 3–21) and was rescored to align with the 4–25 range (consistent with the possible range of the five-item rMEQ). More details can be found in electronic supplementary material, figure S3. The gender analysis only included females and males.

## 3. Results

### (a) Study 1: lifespan

**Figure 1.**
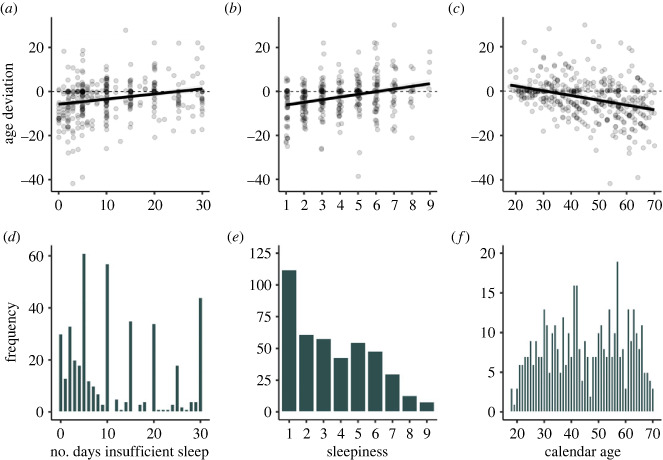
Study 1. (*a*) Regression line of the association between number of days with insufficient sleep in the past 30 days and age deviation in years (subjective age minus calendar age; higher scores indicate feeling older than one's calendar age). (*b*) Regression line between subjective sleepiness (measured by the Karolinska Sleepiness Scale) and age deviation. (*c*) Regression lines between calendar age and age deviation. (*d*) Distribution of number of days with insufficient sleep (range: 0–30). (*e*) Distribution of sleepiness (ranging from 1 extremely alert to 9 extremely sleepy. (*f*) Distribution of calendar age (range: 18–70). Shaded areas represent 95% confidence intervals. Dots represent individual data points.

An increase in the number of days with insufficient sleep and sleepiness was found to be associated with a subjectively older age, figure 1. For each additional day of insufficient sleep during the last 30 days, subjective age increased by 0.23 years (95% CI [0.15, 0.32], *p* < 0.001). For each unit increase in sleepiness on the 9-point KSS scale, subjective age increased by 1.22 years (95% CI [0.87, 1.57], *p* < 0.001). Those who reported having zero out of 30 days of insufficient sleep felt younger than their calendar age, with a subjective age that was on average 5.81 years younger. A KSS score of 1 was associated with a subjective age that was 7.45 years younger than their calendar age. Insufficient sleep remained a significant predictor of subjective age (*b* = 0.14, 95% CI [0.05, 0.23], *p* = 0.003) upon adding sleepiness (*b* = 0.96, 95% CI [0.58, 1.35], *p* < 0.001) to the model. This implies that there are other factors, in addition to sleepiness, that contribute to subjective age. The relationship between subjective age and calendar age showed the expected pattern: as individuals' calendar age increased, they felt progressively younger than one's actual age (*b* = −0.21, 95% CI [−0.27, −0.16], *p* < 0.001). Gender had no significant effect in any of the analyses. The exploratory analyses assessing the influence of chronotype on the relationships between sleep and subjective age (morning types *n* = 111, intermediate types *n* = 239, evening types = 78, missing *n* = 1) indicated that evening types felt 2.14 years older than intermediate types (95% CI [0.04, 4.23], *p* = 0.046) and 2.36 years older than morning types (95% CI [−0.04, 4.77], *p* = 0.054) (adjusted for age; see electronic supplementary material, figure S1). The relationship between subjective age and insufficient sleep was similar across chronotypes, i.e. chronotype did not interact with insufficient sleep (see electronic supplementary material, figure S2).

### (b) Study 2: experimental sleep restriction

**Figure 2.**
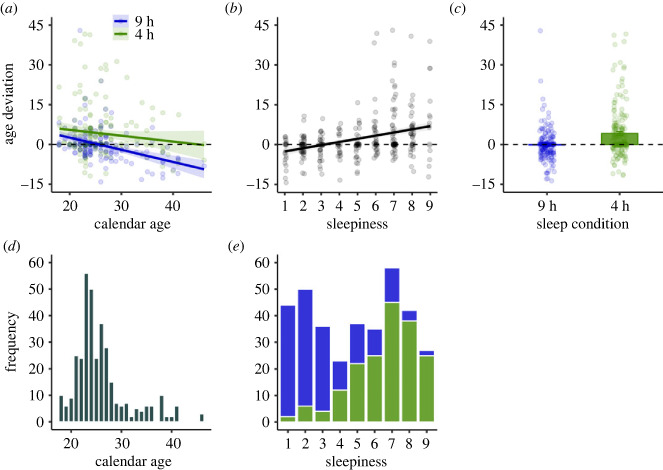
Study 2. (*a*) Regression lines of the relationship between calendar age and age deviation in years (subjective age minus calendar age; higher scores indicate feeling older than their calendar age) for the two sleep conditions: sleep saturation (two nights with 9 h in bed per night) and sleep restriction (two nights with 4 h in bed per night). (*b*) Regression line illustrating the relationship between sleepiness (measured by the Karolinska Sleepiness Scale; higher scores indicate greater sleepiness levels) and age deviation in years. (*c*) The effect of sleep condition on age deviation. (*d*) Distribution of calendar age (ranging from 18 to 46). (*e*) Distribution of sleepiness in the sleep saturation (blue) and sleep restriction (green) condition. Shaded areas and error bars represent 95% confidence intervals. Dots represent individual data points.

After sleep restriction, participants felt on average 4.44 years older as compared to after sleep saturation (95% CI [2.99, 5.90], *p* < 0.001), where they felt 0.24 years younger than their calendar age, figure 2. Sleepiness levels increased with 3.4 scale steps after sleep restriction when compared with sleep saturation (95% CI [3.07, 3.80], *p* < 0.001). Similar to Study 1, for each unit increase in sleepiness on the 9-point KSS scale, subjective age increased by 1.23 years (95% CI [0.94, 1.52], *p* < 0.001), with a KSS score of 1 (extremely alert) being associated with feeling 4.03 years younger than their calendar age. As expected, as individuals' calendar age increased, they felt progressively younger than one's actual age (*b* = −0.33, 95% CI [−0.51, −0.16], *p* < 0.001). The sleep condition effect became non-significant (*b* = 0.47, 95% CI [−1.51, 2.45], *p* = 0.639) after adding sleepiness (*b* = 1.16, 95% CI [0.75, 1.58], *p* < 0.001) to the model, suggesting that sleepiness is a mechanism through which insufficient sleep affects subjective age. Gender had no significant effect in any of the analyses. Exploratory analyses showed interaction effects between chronotype and sleep condition on subjective age, adjusted for age. The sample consisted of 44 morning types, 102 intermediate types and 35 evening types, with five participants having missing rMEQ data. Morning types showed a 5.34 larger increase in subjective age as compared to evening types (95% CI [0.88, 9.79], *p* = 0.019) and 4.02 when compared with intermediate types (95% CI [0.47, 7.58], *p* = 0.027; see electronic supplementary material, figure S4). These interaction effects were largely due to morning types feeling younger in the sleep saturation condition (to evening types *b* = −3.35, 95% CI [−6.08, −0.61], *p* = 0.017 and to intermediate types *b* = −2.95, 95% CI [−5.13, −0.77], *p* = 0.008). Intermediate types and evening types did not significantly differ from each other in the sleep saturation condition (intermediate types as reference: *b* = 0.39, 95% CI [−1.88, 2.66], *p* = 0.732). These results suggest that morning types, on average, feel subjectively younger when sleep is saturated but experience a larger increase in subjective age when exposed to sleep restriction.

## 4. Discussion

Both studies, one cross-sectional and one experimental, demonstrate that sleep and sleepiness play a profound role in shaping our sense of age. The findings revealed that insufficient sleep and sleepiness led people to feel older than their actual age and support that a good night's sleep is central for feeling younger than one's actual age. The analyses also showed that the importance of sleep for subjective age increases with older age, that the relationships are true for both prolonged periods of insufficient sleep and for as little as two nights of insufficient sleep, that sleepiness is a likely mediator for feeling older, and that the relationship between insufficient sleep and feeling older is causal, driven by sleepiness. While the previous literature has shown that feeling older is associated with worse sleep quality [6,8], our data indicate that sleep may be more important for subjective age than the other way around. These findings support that sleep, a vital biological phenomenon, might hold the key to feeling young.

Although sleep serves various recuperative functions [9,11], the biological mechanisms behind its role in influencing subjective age are likely multifaceted and challenging to investigate. This complexity is particularly evident since feeling young is related to multiple aspects including well-being, better brain health and feeling energized. Existing evidence suggests that disturbed sleep could speed up biological ageing, such as telomere shortening and cellular senescence [16] and future studies may further evaluate the extent to which these mechanisms also predict subjective ageing. Sleepiness has previously been shown to strongly influence the motivation to be socially and physically active [17,18], making it a possible mediator through which insufficient sleep reduces the feeling of youthfulness. Given that sleepiness can be readily influenced and altered, interventions involving exposure to daylight or consumption of caffeine could potentially lead to feeling younger. This, in turn, may foster a more active lifestyle and encourage behaviours that promote health.

An important insight from the experimental study is that our perception of age is remarkably malleable. This extends previous research showing that undergoing a memory test can lead individuals to feel older, although in older individuals only [19]. Considering that both sleep and sleepiness are modifiable factors, these discoveries open up new possibilities for fostering a youthful feeling and facilitating associated benefits like a more active lifestyle and embracing new challenges.

We found no gender associations in any of our two studies (one carried out in Sweden and one in the UK), which is in line with some previous findings [1]. This differs from other studies that have found women to feel subjectively younger than men, particularly elderly women [20], although the opposite has also been reported—women feeling subjectively older than men [21,22].

A limitation of the included studies is their focus on only a few sleep health dimensions [23]. The present study primarily focused on how sleep duration, sleep insufficiency and sleepiness influence subjective age, and future studies may assess how other dimensions of sleep health, such as tiredness, fatigue and sleep quality, may also influence subjective age. Exploratory analyses indicated that evening chronotypes felt subjectively older than morning- and intermediate chronotypes, but morning chronotypes reported the largest increase in subjective age following sleep restriction. In future research, manipulations of the timing and quality of sleep, as well as manipulations of fatigue (e.g. through inducing inflammation) or energy levels (e.g. through fasting) could provide further insights into the influences impacting subjective age. Furthermore, Study 1 was carried out during summer and considering that natural light levels are higher during this time of year (reducing sleepiness) compared to late autumn and winter, our sample, as a whole, may have felt younger than during a darker period of the year. In Study 2, data collection spanned the entire year and each participant underwent both sleep conditions within the same season, thereby minimizing the confounding impact of differences in natural light and seasonal variations. Future studies may address how factors such as season, latitude and individual differences in light behaviour may influence subjective age. While the results are robust for a general population sample, future studies are needed to explore the role of sleep in subjective age among clinical groups and to disentangle the involved mechanisms.

To conclude, our studies provide compelling evidence that sleep and sleepiness have a strong impact on how old we feel, and that safeguarding sleep is probably a key factor for feeling young.

## Data Availability

Data and code are available from Dryad [24]. Additional figures are provided in the electronic supplementary material [25].
